# Control quantum evolution speed of a single dephasing qubit for arbitrary initial states via periodic dynamical decoupling pulses

**DOI:** 10.1038/srep43654

**Published:** 2017-03-08

**Authors:** Ya-Ju Song, Qing-Shou Tan, Le-Man Kuang

**Affiliations:** 1 Key Laboratory of Low-Dimensional Quantum Structures and Quantum Control of Ministry of Education, Department of Physics and Synergetic Innovation Center for Quantum Effects and Applications, Hunan Normal University, Changsha 410081, China; 2 College of Physics and Electronic Engineering, Hainan Normal University, Haikou 571158, China

## Abstract

We investigate the possibility to control quantum evolution speed of a single dephasing qubit for arbitrary initial states by the use of periodic dynamical decoupling (PDD) pulses. It is indicated that the quantum speed limit time (QSLT) is determined by initial and final quantum coherence of the qubit, as well as the non-Markovianity of the system under consideration during the evolution when the qubit is subjected to a zero-temperature Ohmic-like dephasing reservoir. It is shown that final quantum coherence of the qubit and the non-Markovianity of the system can be modulated by PDD pulses. Our results show that for arbitrary initial states of the dephasing qubit with non-vanishing quantum coherence, PDD pulses can be used to induce potential acceleration of the quantum evolution in the short-time regime, while PDD pulses can lead to potential speedup and slow down in the long-time regime. We demonstrate that the effect of PDD on the QSLT for the Ohmic or sub-Ohmic spectrum (Markovian reservoir) is much different from that for the super-Ohmic spectrum (non-Markovian reservoir).

Quantum mechanics puts a speed limit to the evolution of quantum systems. The minimal evolution time between two distinguishable states of a quantum system is referred to as quantum speed limit time (QSLT), which determines the maximum speed of dynamical evolution theoretically. Until now, based on Bures angle or relative purity as the distance measure of two distinguishable states, different bounds on the QSLT for both isolated system dynamics^1^,^2^,^3^,^4^,^5^,^6^,^7^ and open system dynamics^8^,^9^,^10^,^11^,^12^,^13^,^14^,^15^,^16^ have been obtained. Generally, there exist two types of the QSLT, i.e., Mandelstam-Tamm bound and Margolus-Levitin bound. A recent review of the literatures on the QSLT is found in ref. 17. The QSLT is originally used to discuss the role of entanglement in the evolution speed, but recent developments extend its applications to tremendous fields, ranging from quantum computation^18^,^19^, quantum communication^20^,^21^, quantum metrology^8^,^22^,^23^,^24^,^25^,^26^ to quantum optimal control^27^,^28^,^29^,^30^,^31^,^32^. In the last two years, further developments include a subtle connection between the QSLT and decoherence^33^,^34^,^35^,^36^, the initial-state role in the QSLT for open dynamics^37^, applications of the QSLT in quantum phase transition^38^.

One should note that, given an actual driving time *τ* for an open system, the QSLT denoted by *τ*_*QSL*_ also indicates the potential capacity for further evolution acceleration. In the situation *τ*_*QSL*_ = *τ*, it means that the evolution is already along the fastest path and possesses no potential capacity for further acceleration. However in the situation *τ*_*QSL*_ < *τ*, the shorter *τ*_*QSL*_, the greater the capacity for potential speedup will be^13^,^14^. Thus, how to induce a shorter QSLT with respect to a fixed driving time gradually becomes a valuable and significative issue. In recent years, several studies have been made to solve this issue. In ref. 12 the authors found the non-Markovian environment can speed up quantum evolution. Subsequently, this theoretically proposed environment-assisted speed-up was realized experimentally in cavity quantum electrodynamics^39^. Meanwhile, it has been suggested that an external classical driving field^40^ and dynamical decoupling pulses^41^,^42^ can be used to accelerate the evolution speed of qubit open systems for specific initial states of the qubits. However, how to control the quantum evolution speed of qubits in an open system for arbitrary initial states is still an open problem.

In this paper, we present a proposal to manipulate the quantum evolution speed of qubits in an open system for arbitrary initial states by employing periodic dynamical decoupling (PDD) pulses^43^. Unlike previous results for qubits in amplitude damping channel^41^ or in X-Y spin-chain environment^42^, in this paper we investigate the influence of PDD pulse sequences on the QSLT of the qubit in a pure dephasing reservoir in both short- and long-time regimes for arbitrary initial states. Here we assume that the qubit is subjected to a zero-temperature dephasing reservoir with Ohmic-type spectra^44^,^45^,^46^. Our findings show that the QSLT of a dephasing qubit under PDD pulses is determined by the initial and final quantum coherence of the qubit, as well as the non-Markovianity of the system during the evolution. Importantly, the final quantum coherence of the qubit and the non-Markovianity of the system can be modulated by the PDD. It is also found that, the dependence of the QSLT on the number of the PDD pulses is sensitive to the spectra type of the reservoir. The case for super-Ohmic spectrum (i.e., non-Markovian reservoir) is much more different from the case for sub-Ohmic or Ohmic spectra (i.e., Markovian reservoir).

## Results

### The model and solution

The system under our consideration is a single qubit (i.e. a two-level system) coupled to a purely-dephasing bosonic reservoir. Under a train of ideal PDD *π* pulses, the total Hamiltonian is (setting *ħ* = 1)


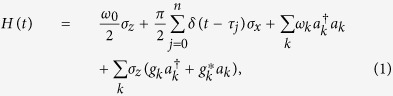


where *ω*_0_ is the qubit frequency, *σ*_*i* = *x*,*y*,*z*_ are the three components of Pauli operators, 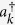 (*a*_*k*_) is the creation (annihilation) operator of the *k*th reservoir mode with the frequency *ω*_*k*_, and *g*_*k*_ is the coupling constant associated with the qubit-*k*th reservoir mode interaction. One should note that the second term of the Hamiltonian in Eq. (1) describes the action of *n* PDD *π* pulses on the qubit within the time *t*. The equally spaced PDD *π* pulses are applied at instants *τ*_*j*_ = (*jτ*_*f*_)/(*n* + 1) with *j* = 1, 2, 3, ……, *n*, and *τ*_*f*_ being the PDD pulses stop time. In fact, from the practical perspective, it is impossible to put infinite PDD pulses on the qubit, and the extra error accumulated by the real imperfect pulses increase with the number of pulses. Consequently, we focus here on the case that the PDD pulses stop at a finite time *τ*_*f*_, after which the system is subjected to the usual decoherence arising from its unavoidable environment^47^.

For the sake of simplicity, we consider the zero-temperature situation and assume that the dephasing reservoir is initially in the vacuum state *ρ*_*B*_ = |0〉_*B*_〈0|, which implies that only the vacuum fluctuation contribution of the reservoir to the qubit decoherence is considered, and the qubit is initially prepared in an arbitrary state


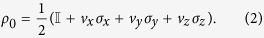


In what follows, we consider the Ohmic-type spectral densities


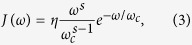


which the Ohmicity parameter *s* is a positive real number. By changing the parameter *s* in Eq. (3), it is possible to go from sub-Ohmic (*s* < 1) to Ohmic (*s* = 1) and *s*uper-Ohmic (*s* > 1) reservoirs, respectively. *η* is a dimensionless coupling constant, and *ω*_*c*_ is a cutoff frequency. Then, in the interaction picture with respect to 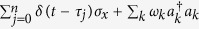 the dynamics of the qubit can be obtained by using the time evolution operator, derived from Eq. (1),


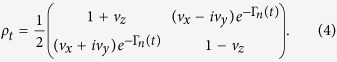


Here the decoherence function under *n* PDD pulses has the form^47^,


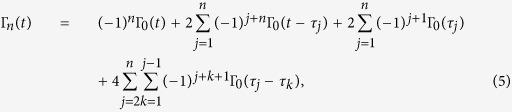


where Γ_0_(*t*) is the decoherence function without DD pulses. The Γ_0_(*t*) for super- and sub- Ohmic spectra can be determined explicitly^23^,


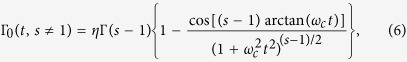


with Γ(*s*−1) being the Euler Gamma function defined as 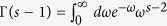. Taking the limit *s* → 1 carefully, one also finds the Γ_0_(*t*) for Ohmic spectrum,


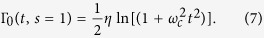


Up to now, the decoherence function under *n* PDD pulses Γ_*n*_(*t*) can be obtained by combining Eqs (6) and (7) with Eq. (5). Significantly, Γ_*n*_(*t*) is not only related to the Ohmic-like spectra, including the Ohmicity parameter *s* and the dimensionless coupling constant *η*, but also can be modulated by the PDD pulses number *n* applied on the qubit within the driving time time *t*. In this way, we arrive at the decoherence function in the whole dynamical process,


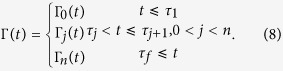


In the following, we will use Γ(*t*) defined as above to study the effect of PDD pulses on the dynamical evolution of the qubit. What is more, from Eq. (4) the master equation for the dynamics of the qubit system can also be obtained as^48^


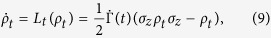


where 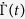 is the first-order derivative of Γ(*t*) in Eq. (8) with respect to *t*, among which the 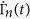 can be derived from Eq. (5),


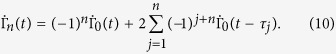


Here 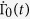 for the super- and sub- Ohmic spectra is given by


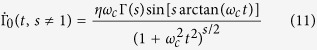


and the 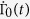 for the Ohmic spectrum


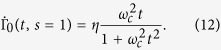


We now pass to the QSLT in order to analyze the effect of DD control on the quantum evolution speed of the dephasing qubit system. As was mentioned in the introduction section, by first fixing the actual driving time *τ, τ*_*QSL*_ = *τ* means that the quantum evolution possesses no potential capacity for further acceleration. While for the case of *τ*_*QSL*_ < *τ*, the shorter *τ*_*QSL*_ indicates the greater speedup potential capacity^13^,^14^. In addition, the QSLT is defined as the minimal time a system needs to evolute from an initial state *ρ*_0_ to a final state *ρ*_*τ*_, which is governed by the time-dependent master equation 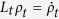. In ref. 15, the authors derived the quantum speed limit bound for arbitrary initial states in the open system based on the von Neumann trace inequality and the Cauchy-Schwarz inequality, which reads


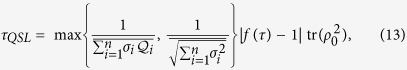


where *σ*_*i*_ and 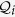 are the singular values of 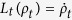 and *ρ*_0_, respectively, 
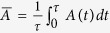
, *f*(*τ*) is the relative purity between the initial state *ρ*_0_ and the final state *ρ*_*τ*_,


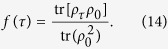


From Eqs (2) and (9), the QSLT can be calculated as the ML-type,


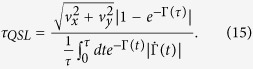


Interestingly, Eq. (15) clearly shows that the QSLT is independent of the qubit initial-state population *v*_*z*_, but it is related to the quantum coherence of the initial state, denoted by 
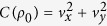
15. It consistently guarantees that without initial coherence, i.e., *C*[*ρ*(0)] = 0, the state of the qubit does not evolve. This means *τ*_*QSL*_ = 0. Furthermore, from Eq. (15) we can conclude that this ML-type bound saturates, i.e., *τ*_*QSL*_ = *τ*, if and only if *C*(*ρ*_0_) = 1 and 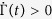 within the driving time *τ*, which is proved to be Markovian process in the following. Note that the saturation implies the dephasing channel connects two states along a geodesic path. To enable us to study the QSLT in details, the non-Markovianity characterized by means of the time evolution of the trace distance is introduced^49^,^50^,^51^,^52^,^53^,


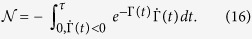


Then, the QSLT can be rewritten as


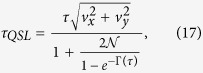


which indicates that the QSLT is not only dependent on the coherence of the initial state *C*(*ρ*_0_), but can also be decreased by the non-Markovianity 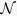 within the evolution time *τ* and the qubit coherence *e*^−Γ(*τ*)^ at the final time *τ*. In fact, the dependence of *τ*_*QSL*_ on the DD pulses is exactly through the Γ(*τ*) and 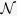. Therefore, one can realize the control of the QSLT for arbitrary initial states through changing the final-state decoherence function Γ(*τ*) and the non-Markovianity function 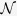 by the use of the PDD pulses.

### PDD-assisted Quantum speedup and slow down

**Figure 1:**
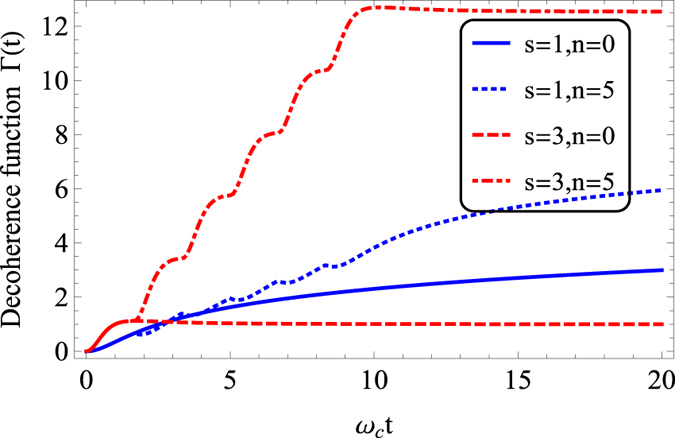
The dynamical evolution of the decoherence function Γ(*t*) under PDD pulses for Ohmic spectrum (*s* = 1) and super-Ohmic spectrum (*s* = 3). The number of PDD pulses is chosen as *n* = 0 (the solid line for the Ohmic reservoir and the dashed line for the super-Ohmic reservoir), or *n* = 5 (the dotted line for the Ohmic reservoir and the dot-dashed line for the super-Ohmic reservoir). Relevant parameters take the value of coupling strength *η* = 0.5, the pulses stop time *τ*_*f*_ = 10. All times are expressed in units of 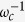.

In order to illustrate the DD-assisted speed control of the single-qubit evolution in the dephasing reservoir, in this section we investigate the influence of PDD pulse sequences number *n* on the QSLT of the qubit system in more detail. It is worth noting that the dynamics of decoherence function Γ(*t*) is sensitive to the spectral parameter *s* in the noise power spectrum *J*(*ω*). As shown in the solid and dashed lines in Fig. 1, without DD pulse (i.e., *n* = 0), the Γ_0_(*t*) monotonically increases with the time in the Ohmic (*s* = 1) reservoir, but the case for the super-Ohmic spectrum (*s* 1) is different: with the increase of time, the Γ_0_(*t*) first increases and then tends to a constant value after a unconspicuous decrease, which results in the phenomenon of coherence trap in the super-Ohmic reservoir^54^. That is to say, without DD pulse, the reservoir is Markovian for the Ohmic spectra, while it is non-Markovian for the super-Ohmic spectrum. Moreover, we find that without DD pulse the reservoir with the sub-Ohmic spectrum is also Markovian, and regarding the use of PDD pulses, the effect of PDD on the QSLT for the sub-Ohmic case is very similar to that for the Ohmic case. Consequently, we will discuss the QSLT only in two kinds of Ohmic-like spectra: the Ohmic spectrum with respect to the Markovian reservoir and the super-Ohmic spectrum with respect to the non-Markovian reservoir. What is more, from Eq. (17) we find the QSLT is also dependent on the actual driving time *τ*. Thus, we will discuss the effect of PDD pulses on the QSLT in both short- and long-time regimes in the following.

### QSLT under PDD pulses in the short-time regime

**Figure 2:**
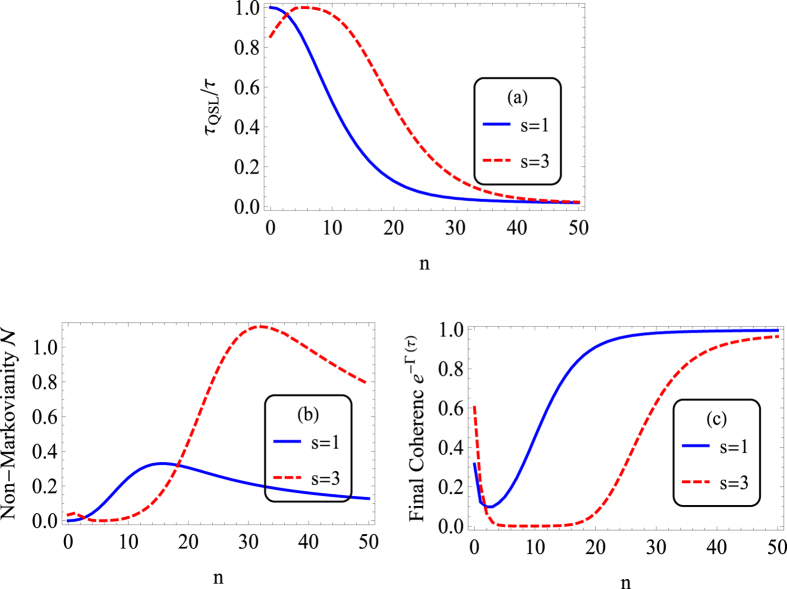
(**a**) QSLT ratio *τ*_*QSL*_/*τ*, (**b**) Non-Markovianity 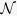 during the driving time *τ*, and (**c**) coherence in the final state 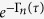 as a function of PDD pulses number *n* in the short-time regime. The solid lines for Ohmic spectrum (*s* = 1, Markovian reservoir), while the dashed lines for super-Ohmic spectrum (*s* = 3, non-Markovian reservoir). Relevant parameters are chosen as *η* = 0.5, 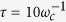, 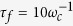, 
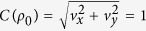
.

We first investigate the effect of PDD pulses on the QSLT in the short-time regime. In Fig. 2(a) is plotted the QSLT ratio *τ*_*QSL*_/*τ* as a function of the PDD pulses number *n* within a given short driving time 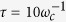. It is clear from the solid line in Fig. 2(a) that the increase of *n* causes a decrease of *τ*_*QSL*_ in Ohmic spectra. In other words, the PDD pulses could induce potential evolution acceleration for the qubit system. In order to illustrate the essential reason behind the acceleration, we plot the Non-Markovianity 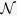 during the driving time *τ* and the coherence at the final state *e*^−Γ(*τ*)^ versus the pulses number *n* in Fig. 2(b,c), respectively. As shown in Eq. (17), the QSLT is determined jointly by above two quantities: 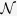 and 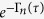. Here the initial coherence has been chosen as 
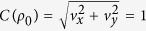
. Inspecting the solid line in Fig. 2(b) we can see that for the Ohmic spectrum, the non-Markovianity 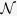 first increases from zero and then decreases with the increase of *n*. This phenomenon may be explained as following: PDD Pulses not only make the effective coupling between qubit and its dephasing reservoir decrease, but also induce the information exchange between them. It is the competition between these two effects that leads to the nonmonotonic behavior of 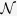 as a function of *n*. Furthermore, we find more PDD pulses would induce greater qubit coherence *e*^−Γ(*τ*)^ except that *n* takes a much small value. In the large *n* limit of *n* → ∞, the value of *e*^−Γ(*τ*)^ even tends to 1. This implies that the decoherence can be suppressed completely if only enough PDD pulses are applied, and the QSLT tends to zero. Combining the solid lines in Fig. 2, we can conclude that for the Ohmic spectrum, the dominant mechanism for the PDD-induced acceleration changes from the non-Markovianity 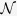 to the coherence *e*^−Γ(*τ*)^ as the pulses number *n* increases.

We pass now to study the PDD-assisted speed control in the short-time regime for the super-Ohmic spectrum. In contrast with the case for the Ohmic spectrum, the case for super-Ohmic spectrum is slightly different when the value of *n* is small. As PDD pulses are added, the *τ*_*QSL*_ has a increase before it begins to decreases. In another word, the qubit system possesses a potential capacity for slow down in the small *n* region and presents a potential speedup in the large *n* region. The transition point from slow down to speedup is about *n* = 5 when the parameters take the value of *η* = 0.5, 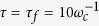. By comparing the dashed lines for super-Ohmic spectrum and solid lines for Ohmic spectrum in Fig. 2, it is not difficult to find that the prime cause of the difference is due to obvious “anti-zeno” effect in the super-Ohmic reservoir, namely the qubit coherence decreases in the small *n* region. For example, the coherence *e*^−Γ(*τ*)^ with *n* = 4 pulses applied is about zero, which is even smaller than that without DD pulses (see the dashed line in Fig. 2(c)). A reasonable explanation for this may also be that the pulses may induced an even faster decoherence if the pulse occurs during a time of re-coherence (information back-flow).

Moreover, if we turn to regard the effect of the coupling constant *η* on the QSLT in Eq. (17), it is found that given a certain *n*, a great *η* would dramatically destroy the qubit coherence and then leads to a change of QSLT. In fact, the dimensionless coupling constant *η*, as the front-factor appearing in Eq. (6) and Eq. (7), could lead to a rescale of the Γ_*n*_(*t*) in Eq. (5) and the 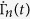 in Eq. (10).

### QSLT under PDD pulses in the long-time regime

**Figure 3:**
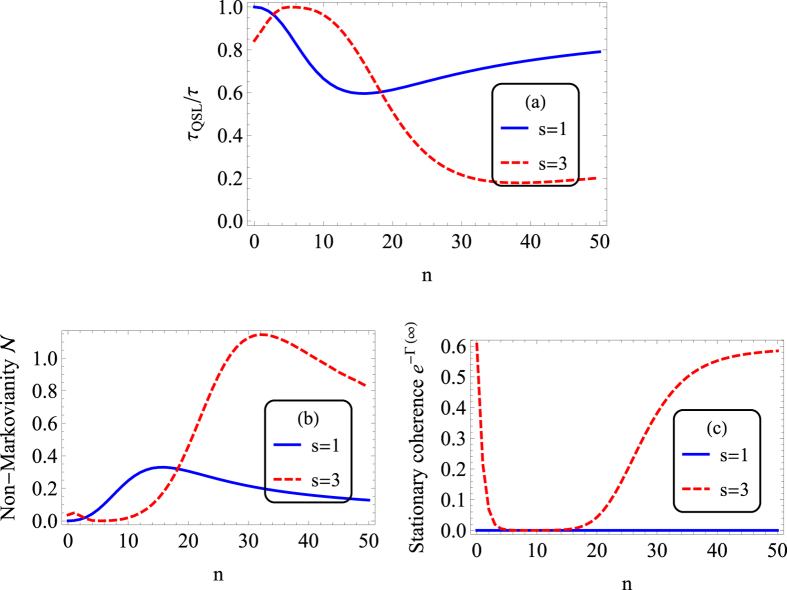
(**a**) QSLT ratio *τ*_*QSL*_/*τ*, (**b**) Non-Markovianity 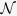 during the driving time *τ*, and (**c**) stationary coherence 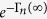 as a function of PDD pulses number *n* in the long-time regime. The solid lines for Ohmic spectrum (*s* = 1, Markovian reservoir), while the dashed lines for super-Ohmic spectrum (*s* = 3, non-Markovian reservoir). Parameters are the same as Fig. 2 except 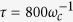.

Let us now look at the QSLT with PDD pulses in the long-time regime. If we chose a sufficiently large fixed driving time *τ*, which ensures the system reaches its steady state, the QSLT *τ*_*QSL*_ reflects the evolution speed of the system relaxing to its steady state. In Fig. 3(a), we consider the relation between the *τ*_*QSL*_/*τ* with the pulses number *n*, when other parameters are the same as that in Fig. 2 except that the driving time here is chosen to be 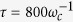.

As far as the asymptotic behavior is considered, the stationary decoherence function can be derived by taking the value of Γ_*n*_(*t*) in Eq. (5) in the long time limit,


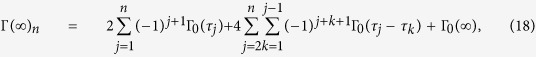


where Γ_0_(*t*) under the zero temperature condition has be given in Eqs (6) and (7). As for the Ohmic reservoir Γ_0_(∞) = ∞, the decoherence function Γ(∞) will also tend to infinite in the long time limit under finite PDD pulses, therefore leading to complete decoherence. Despite this, the case is completely different for the Super-Ohmic reservoir. In the long time limit, both Γ_0_(*t*) and Γ(*t*) for the Super-Ohmic spectrum will tend to a constant value, as a result that DD pulses do not change the dynamic of Γ(*t*) in the long-time regime obviously. Hence we can conclude that the coherence trap only occurs for the super-Ohmic spectrum no matter there exists DD pulses or not.

As we did in the above subsection, we first analyze the effect of PDD pulses on the QSLT for the Ohmic spectrum. In the long-time regime, the QSLT first decreases and then increases as the PDD pulses are added (see the solid line in Fig. 3(a)). That is to say, when the PDD pulses number *n* in the small region (0 < *n* < 12), PDD induces a greater potential capacity for future acceleration of the qubit evolution, but the case is exactly the opposite in the larger *n* region (*n* > 12). The speed control mechanic is that, due to completely decoherence in the long time limit, the QSLT ratio *τ*_*QSL*_/*τ* for Ohmic spectrum is entirely determined by the non-Markovianity, 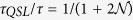. Since the decoherence function Γ(*t*) presents a monotonically increase with respect to the time *t* after the the pulse stop time *τ*_*f*_, the non-Markovianity 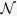 for the long-time regime is exactly determined by the evolution before the time *τ*_*f*_. Because in the short-time regime, we have chosen *τ* = *τ*_*f*_, thus the non-Markovianity 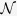 in the long-time regime is exactly the same as that in the short-time regime, as shown in the Figs 2(b) and 3(b). As we have discussed in the above subsection, 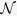 first increases and then declines under PDD pulses, this give rise to the opposite dependence of *τ*_*QSL*_/*τ* on *n* in the long-time regime. In the limit of *n* → ∞, we can easily get *τ*_*QSL*_/*τ* → 1 as a result of 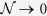.

However, for the super-Ohmic case, the QSLT ratio *τ*_*QSL*_/*τ* in the long-time regime is modulated by the stationary coherence *e*^−Γ(∞)^ and the non-Markovianity 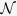 get together, 
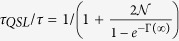
. Now we focus on the effect of PDD pulses on the coherence trap for the Super-Ohmic spectra. Figure 3(c) reports the dependence of stationary coherence *e*^−Γ(∞)^ on the PDD pulse number *n*. Initially we thought that PDD pulses would also enhance the coherence trap in the long-time regime, as they did in the short-time regime. However, a more closer inspection reveals that the use of PDD pulses will destroy the stationary coherence. As *n* increases, *e*^−Γ(∞)^ first declines and then increases, even in the limit of *n* → ∞, the stationary coherence just tends to the value of the zero DD pulses case Γ_0_(∞) = *η*Γ(*s* − 1). The foremost cause of this rather contradictory result is that PDD pulses not only delay the dynamical evolution of Γ(*t*) for about the time *τ*_*f*_, but also make the Γ(*t*) start to monotonically increase from a higher value about 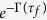 in contrast to the zero DD pulses case, as shown in the dot-dashed line of Fig. 1. The first decrease of *e*^−Γ(∞)^ versus *n* can also be explained by the “anti-zero” effect of 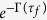 in the short-time regime. While as the PDD pulses number *n* continues to increase, 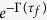 take a lower value, and the destructive effect is weaken. As a result, when PDD pulses are applied, *e*^−Γ(∞)^ is first destroyed seriously and then slowly recovered to the value of *η*Γ(*s* − 1). Besides, with respect to PDD pulses number *n*, the non-Markovianity 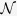 for the super-Ohmic presents a slow decay firstly, and then shows a increases, and finally a decrease again. Consequently, *τ*_*QSL*_/*τ* in the super-Ohmic reservoir first increases slowly, then decreases and finally increases, as a result of the competition between the effects of *n* on 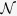 and *e*^−Γ(∞)^.

## Discussion

In summary, we have provided a detailed theoretical study of the application of periodic dynamical decoupling pulse sequences in the evolution speed control for a pure dephasing qubit system. In particular, we have compared the performance of PDD for Ohmic and super-Ohmic spectra in both the short- and long-time regimes. In the short-time regime, despite the “anti-zeno” effect in the very small *n* region, PDD pulses could be used to speedup the evolution of the qubit system in both two kinds of spectra. The QSLT is mainly effected by the non-Markovianity in the small *n* region, but by the qubit coherence in the larger *n* region. In the long-time regime, the cases become more complicated. We find that the stationary coherence only occurs in the super-Ohmic spectrum no matter there are PDD pulses or not. More surprisingly, DD pules would reduce the stationary coherence, and even in the infinite large pulses number limit, the stationary coherence just tends to the value in the zero DD pulses case. Consequently, for the Ohmic spectra, the QSLT is absolutely determined by the non-Markovianity as a result of zero stationary coherence, and it presents first decreases and then increases with more pulses. In other words, the evolution of the qubit relaxing to its steady state possesses first potential acceleration and then slow down. While for the super-Ohmic spectrum, the QSLT first increases, then decreases and finally increases, as a result of the competition between the stationary coherence and non-Markovianity. Furthermore, one should note that the QSLT is also dependent on the initial coherence.

Finally, we would like to aware that our research may have two limitations. The first is that we assume all the PDD pulses are execute quickly and perfectly. Having efficient error correction in mind, we have paid special attention to the case that the PDD pulse stops at a finite time, after which the system is subjected to the usual decoherence arising from its unavoidable environment. The second is the zero-temperature assumption. In order to obtain the analytical expression of the decoherence function, we assume the reservoir is initially prepared at the zero temperature. Noting that the stationary coherence is also sensitive to the temperature of the reservoir, and it could be drastically destroyed by the thermal fluctuations of reservoir. Despite these limitations, we believe our work takes a new look at the use of DD protocol in the evolution speed control for a dephasing qubit system.

## Methods

### The derivation of decoherence function with DDPs

The effect of each ideal PDD *π* pulse on the qubit in Eq. (1) is simply a rotation around the *x* axis by *π*, with transform action 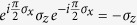 at the time *τ*_*j*_. In the interaction picture with respect to 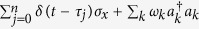, from Eq. (1) we can obtain the effective Hamiltonian


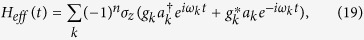


where we have discarded the conserved quantity 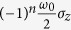. With the help of Magnus expansion^55^ and ignoring an overall phase factor, the time evolution operator can be obtained from Eq. (19),


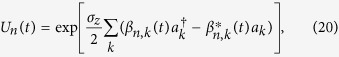


with the DD-dependent displacement parameter being


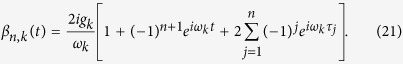


For the sake of simplicity, we assume initially the dephasing reservoir is in the vacuum state *ρ*_*B*_ = |0〉_*B*_〈0|, and the qubit is initially prepared in an arbitrary state 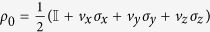. Then, the dynamics of the qubit can be derived as Eq. (4) by using the time evolution operator in Eq. (20). And the decoherence function under *n* PDD pulses has the form


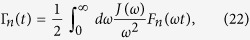


where the noise power spectrum and the filter function are


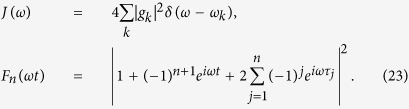


The decoherence function without DD pulses, denoted by Γ_0_(*t*), is similar to Eq. (22) but with *F*_*n*_(*ωt*) replaced by 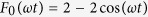,


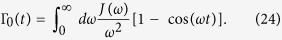


Thus, the relation between Γ_*n*_(*t*) and Γ_0_(*t*), as shown in Eq. (5), can be easily found by rewriting *F*_*n*_(*ωt*) into the form of cosine function^47^. What is more, if we only focus on the Ohmic-like spectra in Eq. (3), the Γ_0_(*t*) can be determined explicitly by inserting Eq. (3) into Eq. (24), as shown in Eqs (6) and (7). Thus, the decoherence function under *n* PDD pulses Γ_*n*_(*t*) can be obtained by combining Eqs (6) and (7) with Eq. (5).

## Additional Information

**How to cite this article:** Song, Y.-J. *et al*. Control quantum evolution speed of a single dephasing qubit for arbitrary initial states via periodic dynamical decoupling pulses. *Sci. Rep.* **7**, 43654; doi: 10.1038/srep43654 (2017).

**Publisher's note:** Springer Nature remains neutral with regard to jurisdictional claims in published maps and institutional affiliations.

## References

[b1] Mandelstam L. & Tamm I. G. The uncertainty relation between energy and time in nonrelativistic quantum mechanics. J. Phys. (USSR) 9, 249–254 (1945).

[b2] Uhlmann A. An energy dispersion estimate. Phys. Lett. A 161, 329 (1992).

[b3] Pfeifer P. How fast can a quantum state change with time? Phys. Rev. Lett. 70, 3365 (1993). 10053850 10.1103/PhysRevLett.70.3365

[b4] Margolus N. & Levitin L. B. The maximum speed of dynamical evolution. Phys. D 120, 188–195 (1998).

[b5] Giovannetti V., Lloyd S. & Maccone L. Quantum limits to dynamical evolution. Phys. Rev. A 67, 052109 (2003).

[b6] Chau H. F. Tight upper bound of the maximum speed of evolution of a quantum state. Phys. Rev. A 81, 062133 (2010).

[b7] Deffner S. & Lutz E. Energy-time uncertainty relation for driven quantum systems. J. Phys. A: Math. Theor. 46, 335302 (2013).

[b8] del Campo A., Egusquiza I. L., Plenio M. B. & Huelga S. F. Quantum speed limits in open system dynamics. Phys. Rev. Lett. 110, 050403 (2013). 23414008 10.1103/PhysRevLett.110.050403

[b9] Carlini A., Hosoya A., Koike T. & Okudaira Y. Time optimal quantum evolution of mixed states. J. Phys. A: Math. Theor. 41, 045303 (2008). 10.1103/PhysRevLett.96.060503 16605975

[b10] Brody D. C. & Graefe E.-M. Mixed-state evolution in the presence of gain and loss. Phys. Rev. Lett. 109, 230405 (2012). 23368172 10.1103/PhysRevLett.109.230405

[b11] Taddei M. M., Escher B. M., Davidovich L. & de Matos Filho, R. L. Quantum speed limit for physical processes. Phys. Rev. Lett. 110, 050402 (2013). 23414007 10.1103/PhysRevLett.110.050402

[b12] Deffner S. & Lutz E. Quantum speed limit for non-Markovian dynamics. Phys. Rev. Lett. 111, 010402 (2013). 23862985 10.1103/PhysRevLett.111.010402

[b13] Xu Z.-Y., Luo S., Yang W.-L., Liu C... & Zhu S. Quantum speedup in a memory environment. Phys. Rev. A 89, 012307 (2014).

[b14] Liu C., Xu Z.-Y... & Zhu S. Quantum-speed-limit time for multiqubit open systems. Phys. Rev. A 91, 022102 (2015).

[b15] Zhang Y.-J., Han W., Xia Y.-J., Cao J.-P. & Fan H. Quantum speed limit for arbitrary initial states. Sci. Rep. 4, 4890 (2014). 24809395 10.1038/srep04890 PMC4013937

[b16] Sun Z., Liu J., Ma J. & Wang X. Quantum speed limits in open systems: non-Markovian dynamics without rotating-wave approximation. Sci. Rep. 5, 8444 (2015). 25676589 10.1038/srep08444 PMC4649631

[b17] Pires D. P., Cianciaruso M., Céeri L. C., Adesso G. & Soares-Pinto D. O. Generalized geometric quantum speed limits. Phys. Rev. X 6, 021031 (2016).

[b18] Warren W. S., Rabitz H. & Dahleh M. Coherent control of quantum dynamics: the dream is alive. Science 259, 1581–1589 (1993). 17733021 10.1126/science.259.5101.1581

[b19] Lloyd S. Ultimate physical limits to computation. Nature 406, 1047–1054 (2000). 10984064 10.1038/35023282

[b20] Bekenstein J. D. Energy cost of information transfer. Phys. Rev. Lett. 46, 623 (1981).

[b21] Yung M.-H. Quantum speed limit for perfect state transfer in one dimension. Phys. Rev. A 74, 030303 (2006).

[b22] Giovannetti V., Lloyd S. & Maccone L. Advances in quantum metrology. Nat. Photonics. 5, 222 (2011).

[b23] Chin A. W., Huelga S. F. & Plenio M. B. Quantum metrology in non-Markovian environments. Phys. Rev. Lett. 109, 233601 (2012). 23368199 10.1103/PhysRevLett.109.233601

[b24] Tsang M. Quantum metrology with open dynamical systems. New J. Phys. 15, 073005 (2013).

[b25] Alipour S., Mehboudi M. & Rezakhani A. T. Quantum metrology in open systems: dissipative Cram&r-Rao Bound. Phys. Rev. Lett. 112, 120405 (2014). 24724633 10.1103/PhysRevLett.112.120405

[b26] Demkowicz-Dobrzański R. & Markiewicz M. Quantum computation speedup limits from quantum metrological precision bounds. Phys. Rev. A 91, 062322 (2015).

[b27] Gordon R. J. & Rice S. A. Active control of the dynamics of atoms and molecules. Annu. Rev. Phys. Chem. 48, 601–641 (1997). 15012451 10.1146/annurev.physchem.48.1.601

[b28] Rabitz H., de Vivie-Riedle R., Motzkus M. & Kompa K. Whither the future of controlling quantum phenomena? Science 288, 824–828 (2000). 10796997 10.1126/science.288.5467.824

[b29] Caneva T. . Optimal control at the quantum speed limit. Phys. Rev. Lett. 103, 240501 (2009). 20366188 10.1103/PhysRevLett.103.240501

[b30] Hegerfeldt G. C. Driving at the quantum speed limit: optimal control of a two-level system. Phys. Rev. Lett. 111, 260501 (2013). 24483786 10.1103/PhysRevLett.111.260501

[b31] Avinadav C., Fischer R., London P. & Gershoni D. Time-optimal universal control of two-level systems under strong driving. Phys. Rev. B 89, 245311 (2014).

[b32] Deffner S. Optimal control of a qubit in an optical cavity. J. Phys. B: At. Mol. Opt. Phys. 47, 145502 (2014).

[b33] Mukherjee V. . Speeding up and slowing down the relaxation of a qubit by optimal control. Phys. Rev. A 88, 062326 (2013).

[b34] Marvian I. & Lidar D. A. Quantum speed limits for leakage and decoherence. Phys. Rev. Lett. 115, 210402 (2015). 26636833 10.1103/PhysRevLett.115.210402

[b35] Dehdashti Sh., Harouni M. B., Mirza B. & Chen H. Decoherence speed limit in the spin-deformed boson model. Phys. Rev. A 91, 022116 (2015).

[b36] Brouzos I. . Quantum speed limit and optimal control of many-boson dynamics. Phys. Rev. A 92, 062110 (2015).

[b37] Wu Sh.-X., Zhang Y., Yu C.-S. & Song H.-S. The initial-state dependence of quantum speed limit. J. Phys. A: Math. Theor. 48, 045301 (2015).

[b38] Wei Y.-B., Zou J., Wang Z.-M. & Shao B. Quantum speed limit and a signal of quantum criticality. Sci. Rep. 6, 19308 (2015). 10.1038/srep19308 PMC4725993 26782296

[b39] Cimmarusti A. D. . Environment-assisted speed-up of the field evolution in cavity quantum electrodynamics. Phys. Rev. Lett. 114, 233602 (2015). 26196802 10.1103/PhysRevLett.114.233602

[b40] Zhang Y.-J., Han W., Xia Y.-J., Cao J.-P. & Fan H. Classical-driving-assisted quantum speed-up. Phys. Rev. A 91, 032112 (2015).

[b41] Song Y.-J., Kuang L.-M. & Tan Q.-S. Quantum speedup of uncoupled multiqubit open system via dynamical decoupling pulses. Quantum Inf. Process. 15, 2325 (2016).

[b42] Wei Y.-B., Zou J., Wang Z.-M., Shao B. & Li H. Dynamical decoupling assisted acceleration of two-spin evolution in XY spin-chain environment. Phys. Lett. A 380, 397 (2016).

[b43] Gullion T., Baker D. B. & Conradi M. S. New, compensated Carr-Purcell sequences. J. Magn. Reson. 89, 479–484 (1990).

[b44] Kuang L.-M., Zeng H.-S. & Tong Z.-Y. Nonlinear decoherence in quantum state preparation of a trapped ion. Phys. Rev. A 60, 3815 (1999).

[b45] Kuang L.-M., Ouyang Z.-W., Tong Z.-Y. & Zeng H.-S. Decoherence in two Bose-Einstein condensates. Phys. Rev. A 61, 013608 (1999).

[b46] Yuan J.-B. & Kuang L.-M. Quantum-discord amplification induced by a quantum phase transition via a cavity¨CBose-Einstein-condensate system. Phys. Rev. A 87, 024101 (2013).

[b47] Addis C., Ciccarello F., Cascio M., Palma G. & Maniscalco S. Dynamical decoupling efficiency versus quantum non-Markovianity. New J. Phys. 17, 123004 (2015).

[b48] Breuer H.-P. & Petruccione F. The Theory of Open Quantum Systems (Oxford University Press, New York, 2002).

[b49] Breuer H.-P., Laine E.-M. & Piilo J. Measure for the degree of non-Markovian behavior of quantum processes in open systems. Phys. Rev. Lett. 103, 210401 (2009). 20366019 10.1103/PhysRevLett.103.210401

[b50] Laine E.-M., Piilo J. & Breuer H.-P. Measure for the Non-Markovianity of quantum processes. Phys. Rev. A 81, 062115 (2010).

[b51] Wissmann S., Karlsson A., Laine E.-M., Piilo J. & Breuer H.-P. Optimal state pairs for non-Markovian quantum dynamics. Phys. Rev. A 86, 062108 (2012).

[b52] HeZ., ZouJ., LiL. & ShaoB. Effective method of calculating the non-Markovianity 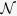 for single-channel open systems. hys. Rev. AP 83, 012108 (2011).

[b53] Xu Z.-Y., Yang W.-L. & Feng M. Proposed method for direct measurement of the non-Markovian character of the qubits coupled to bosonic reservoirs. Phys. Rev. A 81, 044105 (2010).

[b54] Addis C., Brebner G., Haikka P. & Maniscalco S. Coherence trapping and information backflow in dephasing qubits. Phys. Rev. A 89, 024101 (2014).

[b55] Blanes S., Casas F., Oteo J. A. & Ros J. The Magnus expansion and some of its applications. Phys. Rep. 470, 151–238 (2009).

